# The potent anti-inflammatory effect of Guilu Erxian Glue extracts remedy joint pain and ameliorate the progression of osteoarthritis in mice

**DOI:** 10.1186/s13018-018-0967-y

**Published:** 2018-10-19

**Authors:** Yen-Jung Chou, Jiunn-Jye Chuu, Yi-Jen Peng, Yu-Hsuan Cheng, Chin-Hsien Chang, Chieh-Min Chang, Hsia-Wei Liu

**Affiliations:** 1 Department of Traditional Chinese Medicine, MacKay Memorial Hospital, Taipei City, Taiwan; 2 Department of Life Science, Fu Jen Catholic University, New Taipei City, Taiwan; 3 Graduate Institute of Applied Science and Engineering, Fu Jen Catholic University, No. 510, Zhongzheng Rd., Xinzhuang Dist., New Taipei City, 24205 Taiwan; 4 Department of Biotechnology, College of Engineering, Southern Taiwan University, Tainan City, Taiwan; 5 Department of Pathology, Tri-Service General Hospital, National Defense Medical Center, Taipei City, Taiwan; 6 Department of Cosmetic Science, Chang Gung University of Science and Technology, Tao-Yuan City, Taiwan; 7 Department of Traditional Chinese Medicine, En Chu Kong Hospital, New Taipei City, 237 Taiwan

**Keywords:** Osteoarthritis, Anterior cruciate ligament, Guilu Erxian Glue, Knee, Articular cartilage, Mice

## Abstract

**Background:**

Osteoarthritis (OA) is a slow progressing, degenerative disorder of the synovial joints. Guilu Erxian Glue (GEG) is a multi-component Chinese herbal remedy with long-lasting favorable effects on several conditions, including articular pain and muscle strength in elderly men with knee osteoarthritis. The present study aimed to identify the effects of Guilu Erxian Paste (GE-P) and Liquid (GE-L) extracted from Guilu Erxian Glue in anterior cruciate ligament transection (ACLT)-induced osteoarthritis mice, and to compare the effectiveness of different preparations on knee cartilage degeneration during the progression of osteoarthritis.

**Methods:**

Male C57BL/6J mice underwent anterior cruciate ligament transection to induce mechanically destabilized osteoarthritis in the right knee. 4 weeks later, the mice were orally treated with PBS, celecoxib (10 mg/kg/day), Guilu Erxian Paste (100 or 300 mg/kg/day), and Guilu Erxian Liquid (100 or 300 mg/kg/day) for 28 consecutive days. Von Frey and open-field tests (OFT) were used to evaluate pain behaviors (mechanical hypersensitivity and locomotor performance). Narrowing of the joint space and osteophyte formation were examined radiographically. Inflammatory cytokine (IL-1β, IL-6, and TNF-α) levels in the articular cartilage were determined by quantitative real-time PCR. Histopathological examinations were conducted to evaluate the severity and extent of the cartilage lesions.

**Results:**

Guilu Erxian Paste and Guilu Erxian Liquid (300 mg/kg/day) were significantly more effective (*p* < 0.01) than celecoxib (10 mg/kg/day) in decreasing secondary allodynia when compared to the saline-treated group (^#^*p* < 0.05). Open-field tests revealed no significant motor dysfunction between the Guilu Erxian Paste- and Guilu Erxian Liquid-treated mice compared to the saline-treated mice. Radiographic findings also confirmed that the administration of Guilu Erxian Paste and Guilu Erxian Liquid (100 and 300 mg/kg/day) significantly and dose-dependently reduced osteolytic lesions and bone spur formation in the anterior cruciate ligament transection-induced osteoarthritis mice when compared to the saline-treated group. Notably, Guilu Erxian Liquid (100 mg/kg/day) treatment significantly reduced the mRNA levels of IL-1β, IL-6, and TNF-α as well as relative the protein expression of IL-1β and TNF-α to the effect of celecoxib. Guilu Erxian Paste and Guilu Erxian Liquid (300 mg/kg/day) markedly attenuated cartilage destruction, surface unevenness, proteoglycan loss, chondrocyte degeneration, and cartilage erosion in the superficial layers (^##^*p* < 0.01 and ^###^*p* < 0.001 respectively)**.**

**Conclusions:**

As expected, our findings suggest that the anti-inflammatory effects of Guilu Erxian Liquid (GE-L), following marked decrease on both IL-1β and TNF-α during the early course of post-traumatic osteoarthrosis (OA), may be of potential value in the treatment of osteoarthritis.

## Background

Osteoarthritis (OA), the most frequent chronic musculoskeletal disorder, is a slowly progressing disease characterized by articular cartilage degeneration, subchondral bone changes, osteophyte formation, low-grade synovial inflammation, and hypertrophic bone changes, leading to pain and functional deterioration [1, 2] . It is the most prevalent form of disease that involves cartilage degradation and periarticular bone responses, especially in the knee [3]. OA can affect every joint in the body; approximately 10–12% of the adult population is affected by this disease [4]. Individuals above 60 years of age present with the pathological features of OA in at least one joint, thus influencing the quality of life and resulting in enormous costs to the healthcare system [5]. Although OA is one of the most common disorders of the joints with a rising prevalence, it is difficult to treat the disease using current therapies [6, 7]. Given the complexity of this pathology, there are no pharmaceutical treatments that can slow the disease progression due to the limited knowledge about the pathogenesis of this condition [8, 9].

In the joint, the tissues containing nociceptors include primarily the joint capsule, ligaments, synovium, bone, and the outer edge of the menisci (in the knee) [10–12]. Inflammation lowers the threshold for nociception; while cytokines are being assessed as possible candidates for biochemical markers, inflammation is increasingly being considered as an important part of the pathophysiology of OA [13–15]. According to the American College of Rheumatology 2000 guidelines, patients with OA of the knee, a condition characterized by cartilage degradation, are often treated with steroids, non-steroidal anti-inflammatory drugs (NSAIDs), and cyclooxygenase-2 selective NSAIDs (e.g., celecoxib), which relieve pain and inflammation but are not capable of restoring tissues once OA has initiated [16, 17].

OA is a chronic progressive disease with complicated mechanisms that include inflammation, periarticular bone response, and cartilage degradation [18–20]. To date, the available pain treatments are limited in their efficacy and known to possess associated toxicities, none of which halts disease progression or regenerates damaged cartilage or bone [21]. Thus, current therapeutic strategies seek to ameliorate pain, offer chondroprotective or regenerative capability, and increase mobility, thereby representing the critically unmet needs. Notably, the anti-inflammation, anti-apoptosis, and anti-catabolism activities of several traditional Chinese medicines (TCM) provide a proposed medical option by modifying the disease and its symptoms in OA [22] .

Guilu Erxian Glue (GEG), which comprises four major components, *Testudinis Plastrum*, *Cornu Cervi*, *Lycii Fructus*, and *Ginseng Radix*, is a Chinese herbal remedy that has long-lasting favorable effects on aging, perimenopausal syndrome, and degenerative joint diseases in Asia [23–25]. Recent studies have also mentioned that GEG can stimulate the secretion of IGF-1 in osteoblasts and attenuate the bone resorption activity of osteoclasts in vitro [26], inhibit the formation of osteoclasts and bone pits in rats [27], and decrease articular pain and increase muscle strength in elderly men with knee OA [28]. However, knowledge about the mechanisms responsible for the beneficial effects of GEG in OA is limited.

Post-traumatic arthritis by bilateral transection of the anterior cruciate ligament (ACLT) is one of the most frequent causes of disability following joint trauma [29, 30]. Hence, in the present study, we developed a post-traumatic OA mouse model to investigate the pathophysiology of knee OA and the molecular characteristics of knee joint cartilage. During the course of the experiments, tests for mechanical hypersensitivity (von Frey test) and locomotor performance (open-field test) were used to evaluate pain behaviors associated with ACLT-induced OA mice. The present study aimed to identify the anti-nociceptive and anti-inflammatory effects of Guilu Erxian Paste (GE-P) and Guilu Erxian Liquid (GE-L) extracted from different preparations of GEG in ACLT-induced OA mice.

## Methods

### Chemicals and reagents

Celecoxib was produced by Pfizer Inc. (Manhattan, NY, USA), and Ketoprofen was produced by Swiss Co., Ltd. (Xinshi, TNN, Taiwan). Zoletil was purchased from Virbac (Grasse, Carros, France). Hematoxylin and eosin (HE), xylene, and paraffin were purchased from Thermo Fisher Scientific Inc. (Waltham, MA, USA). Safranin-O/fast green histochemical stain was obtained from ScienCell Research Laboratories. (Carlsbad, CA, USA). Designed Real-Time RT-PCR Primers (GAPDH, IL-6, IL-1β, and TNF-α) were ordered from Integrated DNA Technologies (Coralville, IA, USA). Phenol-Free Total RNA Purification Kit was purchased from AMRESCO (Solon, IA, USA). SYBR Green was ordered from Protech Technology Enterprise Co., Ltd. (Nangang, TPE, Taiwan). The rabbit polyclonal antibodies-IL-1β and TNF-α, HRP-anti-rabbit IgG, and HRP anti-mouse IgG were from Santa Cruz Biotechnology, Inc. (Delaware, California, USA). Guilu Erxian Paste (GE-P) and Guilu Erxian Liquid (GE-L) were prepared (100 and 300 mg/kg/day, respectively) for treating the ACLT-induced OA mice.

### Animals

Male C57BL/6J mice, 9 weeks of age (10 weeks at time of injury), were purchased from the National Laboratory Animal Center, Taiwan. All animals were maintained in laminar flow cabinets with free access to food and water under specific pathogen-free conditions in facilities approved by the Accreditation of Laboratory Animal Care and in accordance with the Institutional Animal Care and Use Committee of the Animal Research Committee of the Southern Taiwan University of Science and Technology, Tainan, Taiwan. Five mice per cage were fed with mouse chow and water ad libitum. The mice were acclimatized to the 12/12-h light–dark cycle conditions in the cages and were kept in the housing facility for a 1-week acclimation period before surgical injury.

### Preparation of Guilu Erxian Glue extracts

The herbs and extract, GE-P, and GE-L, were prepared by the Taiwan Herbal Biopharma Co., Ltd., which is a TCM Good Manufacturing Practice manufacturer certified in Taiwan. GEG was comprised of the following medicinal herbs: *Testudinis Plastrum*, *Cornu Cervi*, *Lycii Fructus*, and *Ginseng Radix* at a weight proportion of 10:5:1.3:1, sequentially. According to the well-documented TCM formula described in a TCM book known as “The Golden Mirror of Medicine.” GE-P was prepared by stewing *Testudinis Plastrum* and *Cornu Cervi* for 7 days, after which, *Lycii Fructus* and *Radix Ginseng* were added to the mixture, filtered, and a concentrated paste was formed. GE-L was prepared by conventional hot-water reflux extraction concentrated under reduced pressure; 1 kg of GE-P was dissolved in 5 L of double-distilled water and extracted with hot water (70 °C) after 8–9 h. The impurity-free solutions were stored at − 80 °C until use. The working concentrations of GE-P and GE-L were determined by calculating the initial weight of the raw materials (g) and the final vehicle volume (mL). High-performance liquid chromatography was used to detect the contents in the GE-P samples at 255 ± 19 mg/mL for Putrescine, 6.0 ± 0.8 mg/mL for Scopoletin, and 83 ± 5.3 mg/mL for Ginsenoside Re, respectively. The three compounds were deduced by comparing the individual peak retention times with those of the authentic reference substances. GE-L was also adjusted to the quality (standard effective components) of GE-P in order to prepare the same working concentration for the treatments.

### Osteoarthritis surgery and experimental groups

9-week-old mice were anesthetized with Zoletil, and the anterior cruciate ligament was surgically transected to induce mechanical destabilization on the right knee, causing joint instability and post-traumatic OA. All surgical procedures were performed using a stereoscopic microscope (SMZ1000, Nikon, Tokyo, Japan). 4 weeks after ACLT surgery to restore, all tested mice were forced to run on a treadmill at a speed of 16 m/min every day for 30 consecutive days to achieve the histological progression of the knee OA. Sham operations were conducted by making a capsular incision and subjecting the mice to the treadmill run. The mice were randomized into the following groups: group 1 (*n* = 10), animals underwent sham operations and were orally treated with PBS; group 2 (*n* = 10), animals underwent ACLT surgery with oral vehicle treatment; and group 3 (*n* = 10), animals underwent ACLT surgery and were orally treated with celecoxib (10 mg/kg/day); group 4 (*n* = 10), animals underwent ACLT surgery and were orally treated with GE-P (100 mg/kg/day); group 5 (*n* = 10), animals underwent ACLT surgery and were orally treated with GE-P (300 mg/kg/day); group 6 (*n* = 10), animals underwent ACLT surgery and were orally treated with GE-L (100 mg/kg/day); and group 7 (*n* = 10), animals underwent ACLT surgery and were orally treated with GE-L (300 mg/kg/day). The oral administrations were performed for 28 consecutive days. Celecoxib, a COX-2 selective NSAID, was used as a positive control to evaluate the analgesic and anti-inflammatory roles in OA. The operated mice were sacrificed after the last treatment; at least six mice were used at each time-point for every set of experiments. Body weight was recorded regularly at 0, 2, and 4 weeks after the treatments.

### Tactile sensitivity testing

Prior to pain testing, the mice were habituated to the testing chambers (plexiglass cubicles with a mesh floor) 1 week prior to baseline readings. Subsequently, the animals were acclimated for 30 min on a wire grid platform in individual chambers, before the von Frey testing. Mechanical sensitivity was measured by determining the hind paw withdrawal threshold using a set of 17 von Frey filaments (Somedic SenseLab, Sösdala, Skåne län, Sverige) with ascending force intensities. The force required to buckle the monofilament increases from 0.026 g in the first handle of the set to 110 g in the last (corresponding to a pressure range from 5 g/mm^2^ to 178 g/mm^2^). For optimum accuracy, each case was equipped with a thermo- or hygrometer. Mice were assessed three times at each time-point, and percent changes from baseline readings were reported. A positive response was defined as a rapid withdrawal of the hind paw when the stimulus was applied, and the number of positive responses for each stimulus was recorded. Tactile threshold was defined as a withdrawal response to a given stimulus intensity in 5 of 10 trials. This threshold was calculated once per animal.

### Open-field behavioral testing

Quantitative motor testing was performed to find out whether the nerve pain had resulted from knee OA or from peripheral neuropathy in the mice. Thus, the ambulatory activity was measured using an OFT. All mice were allowed to adapt to the environment for 1 h prior to testing. Mice were placed individually in the center of a square open field (50 × 50 × 50 cm) with white plexiglass walls and were observed for 10 min under normal lighting. Movements and trajectories of the mice were videotaped and analyzed by the TM-01 Animal Video Behavior System (Diagnostic & Research Instruments Co., Ltd., Taiwan), which is a versatile video tracking system for automatically recording and analyzing animal activity, movement, and behavior. All data of given parameters such as motion tracking trajectory and mean velocity (MV) in all four 10 × 10 cm^2^ corners were recorded and calculated by the same TM-01 Video Tracking Software program.

### Radiographic assessments

After 4 weeks of administration, the animals that underwent sham operation or ACLT surgery were anesthetized, and X-rays were taken using a Faxitron Specimen Radiography System (Field Emission Corp., McMinnville, OR). The mice were radiographed at the same exposure for accurate comparisons of the hind legs; lateral radiographs were taken for both experimental and sham groups. The radiographs were analyzed for the presence of bony lesions, fracture callus, and bone remodeling. To facilitate congruency between radiographs, the mice were placed in a prone position with hips in abduction, causing external rotation of the tibia and creating a lateral position. Standardized radiographs of the entire skeleton of the mice were collected at 32 kV with an exposure time of 48 s on manual mode.

### RNA isolation and quantitative real-time PCR analysis

RNA from articular cartilage tissues was isolated using a Phenol-Free Total RNA Purification Kit (Solon, IA, USA), and cDNA was synthesized using iScript reverse transcriptase kit (Bio-Rad Laboratories, Hercules, CA, USA). Quantitative real-time PCR (qPCR) was performed using the Applied Biosystems® 7500 Real-Time PCR Systems (Thermo Fisher Scientific Inc.). The resulting cDNAs were used to assay gene expression using the following primers: IL-6, (Plus: 5′-CAAATTCGGTACATCCTCGAC-3′/Minus:5′-CTACGTTATTGGTGGGGACTG-3′); IL-1β (Plus: 5′-TCA AAG CAA TGT GCT GGT GC-3′/Minus: 5′-ACC TAG CTG TCA ACG TGT GG-3′); TNF-α (Plus: 5′-CGC GGA TCA TGC TTT CTG TG-3′/Minus: 5′-GGA CTA GCC AGG AGG GAG AA-3′); and the housekeeping gene GAPDH (Plus: 5′-GAG CTA CGT GCA CCC GTA AA-3′/Minus: 5′-CAA AAA TGA GGC GGG TCC AA-3′). All reactions were performed using qPCR™ Core Kit for SYBR Green I®. Data were analyzed using the 2^−ΔΔCt^ method followed by its validation. Each experiment was performed in triplicate and repeated at least three times.

### Western blotting analysis

Western blotting was used to evaluate the IL-1β and TNF-α protein expression. Proteins from each articular cartilage were extracted by using lysis buffer [50 mm Tris–HCl, pH 6.8; 10% sodium dodecyl sulfate (SDS)] and homogenized. After 30 min of incubation at 4 °C, the lysates were heated at 100 °C during 5 min and were centrifuged at 10 000×*g* for 30 min at 4 °C. Lysates containing equal amounts of proteins (100 μg) were resolved on 8.5% SDS-polyacrylamide gel electrophoresis. Rainbow-colored protein molecular weight standards obtained from Amersham were used for the estimation of molecular size. The proteins were blotted to a hybond-enzyme chemio luminiscence (ECL) nitrocellulose membrane that was probed and washed according to the instructions for the enhanced chemiluminescence western blotting detection system (Amersham Pharmacia Biotech, Little Chalfont, UK), with transfer buffer (pH 8.3) containing 20% methanol (*v*/*v*) using an Hoefer miniEV electrotransfer unit (Amersham Pharmacia Biotech). The membrane with transferred proteins was blocked with 5% serum albumin in tris-buffered saline (TBS) containing 0.1% Tween 20 (TBST) for 1 h at room temperature and incubated with the first antibody diluted in TBST for 1 h at room temperature. After blocking, membranes were incubated for 1 h at room temperature in wash buffer with either anti-IL-1β antibody (1:1000) and anti-TNF-α antibody (1:1000) followed by four times 10 min washing. Horse radish peroxidase-conjugated anti-rabbit and anti-mouse IgG antibody was diluted to 1:5000 in washed buffer and incubated with blots for 1 h at room temperature. For measuring immunoreactive expression of IL-1β and TNF-α proteins from kidney, finally joins HRP to assume the stain (Reagent A + Reagent B by 1: 1 proportion) on NC membrane under the room temperature responded 1 min develops again using the cold light image analyzer (FUJIFILM LAS-3,000). For quantification of immunoblots, relative intensities of bands were quantified by densitometry using image master image analysis software (Amersham Pharmacia Biotech). Control for loading and transfer was obtained by probing with anti-β-actin.

### Histological evaluation

The mice were sacrificed and the knees were excised; samples were harvested and fixed in 4% paraformaldehyde, decalcified in 9% formic acid for 3–5 days, and embedded in paraffin. The formalin-fixed tissues were sliced into 5-μm-thick sections using a Microtome RM2135 (Leica Microsystems Inc., Bannockburn, IL, USA), placed on to silane-coated slides and immersed in tris-buffered saline, pH 7.4. After rehydration in graded ethanol solutions, the samples were dried overnight at 37 °C, and stored at room temperature. Serial sections of the knees were stained with HE (Sakura Finetek, Tokyo, Japan) and Safranin-O with fast green counterstain. HE staining of OA cartilage (pale blue color) generally indicates extensive cartilage destruction and calcification of the cartilage tissue. A necessary constraint on the validity of this scoring system is the consistency with which cartilage lesions are classified by HE staining. The 14-point Mankin score for evaluation of OA cartilage has also been used for the grading of animal cartilage. Safranin-O, an indicator of cell chondrogenesis, is a cationic dye that stains acidic proteoglycan present in the cartilage tissues. Safranin-O binds to glycosaminoglycan and appears orange-red in color, enabling the assessment of the structural integrity of the cell–extracellular matrix in cartilaginous tissue. Each sample was also stained with Safranin-O/fast green for the histopathological classification of cartilage degeneration during OA progression. The OA grade is defined as an index of the severity or biologic progression of the OA lesion based on the extent of pathology in the cartilage and the destabilization of the medial meniscus. The values range from 0 (surface intact) to 24(full-thickness loss of cartilage and bone deformation) on the OARSI score [31]. Lastly, the slides were examined using a Motic BA 400 microscope with Motic Advance 3.0 software (Motic Co., Fujian, China).

### Theory/calculation

All results are presented as the mean ± standard deviation (SD). Differences between groups were evaluated by analysis of variance and post hoc comparisons with Bonferroni step-down (Holm) correction. Statistical analysis was performed using SigmaPlot software (version 10.0; SPSS Inc., USA). Post hoc testing of behavioral data utilized a two-tailed Welch’s 푡 test. Post hoc testing of biochemical data utilized a regression analysis. Each value represents the mean ± SD from 10 mice. *p* values less than 0.05 were considered statistically significant. ^∗^*p* < 0.05, ^∗∗^*p* < 0.01, and ^∗∗∗^*p* < 0.001 represent significant differences from the sham group (no ACLT). ^#^*p* < 0.05, ^##^*p* < 0.01, and ^###^*p* < 0.001 represent significant differences from the saline group (ACLT).

## Results

### GE extracts attenuate joint pain without affecting motor activity in OA mice

**Fig. 1:**
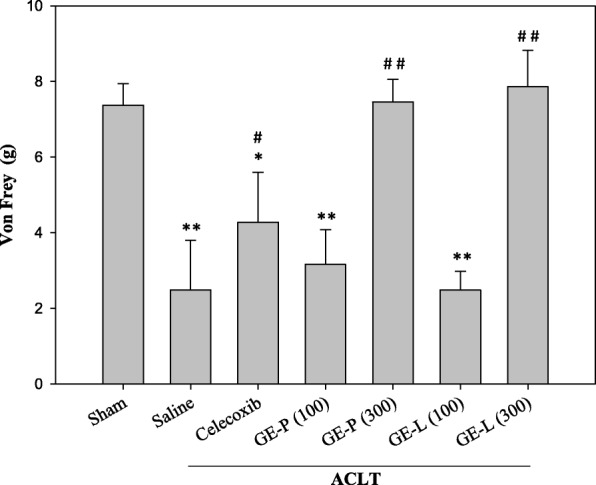
Tactile sensitivity in ACLT-induced OA-related allodynia. Von Frey testing was used to measure the response evoked by mechanical stimulus in the groups treated with GE-P (100 and 300 mg/kg/day), GE-L (100 and 300 mg/kg/day), and celecoxib (10 mg/kg/day) when compared to the saline group. Mechanical hyperalgesia was tested by observing the changes in tactile sensitivity (pain behavior) on day 28, before sacrifice. Paw withdrawal threshold (PWT) was significantly reduced in the saline-treated ACLT mice (**p < 0.01) when compared with the vehicle controls. Each value represents the mean values obtained from 10 mice in two different experimental sets. ^*^p < 0.05 and ^**^p < 0.01 denote significant differences when compared with the vehicle control (sham). ^#^*p* < 0.05 and ^##^*p* < 0.01 denote significant differences when compared with the saline-treated (ACLT) mice

**Fig. 2:**
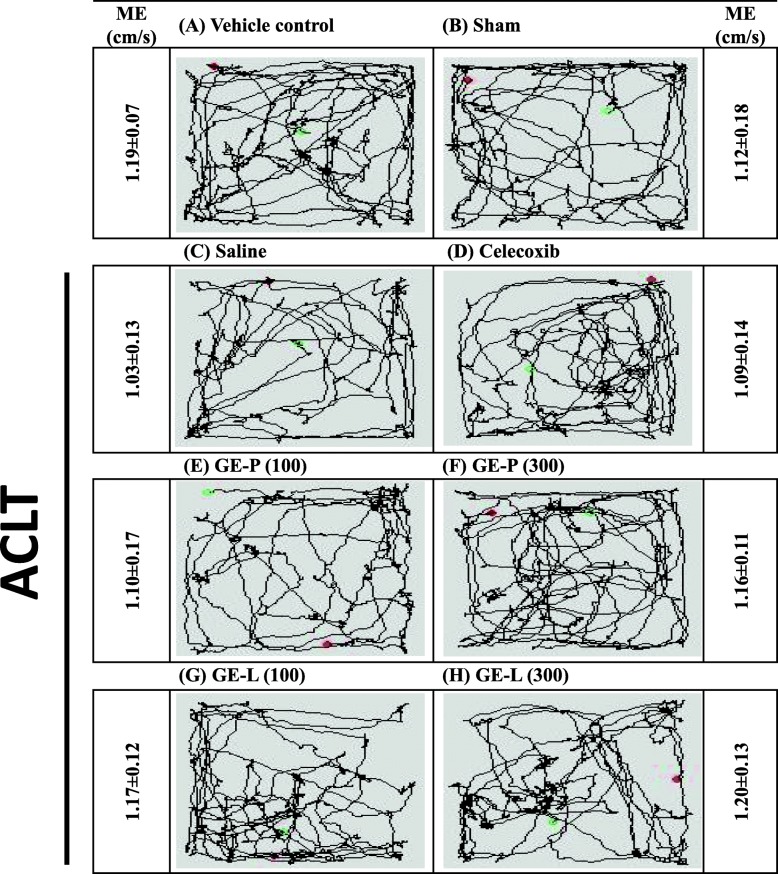
Exploratory assessments in ACLT-induced OA-related pain behavior. There was no ACLT surgery in vehicle control (**a**) and sham (**b**) group. The effects of GE-P (100 mg/kg/day, **e**), GE-P (300 mg/kg/day, **f**), GE-L (100 mg/kg/day, **g**), GE-L (300 mg/kg/day, **h**), and celecoxib (10 mg/kg/day, **d**) when compared with the saline group (**c**) were analyzed by the open-field test (OFT) on day 28, before sacrifice. Each mouse was placed in the corner of an “open field” and allowed to roam the field. Movements were automatically recorded within 10 min via a TM-01 Animal Video Behavior System. Representative images (motion tracking behavior) showing the mouse traveling patterns were obtained and total ambulatory distance during the 10 min were measured by mean velocity (MV). Each value/error bar is expressed as the mean ± SD of 10 mice

The progression of OA is accompanied by secondary clinical symptoms, with pain being the most prominent. In von Frey testing, a non-noxious stimulus was used to measure the response evoked by a mechanical stimulus. We estimated the effects of the GEG extracts GE-P and GE-L on pain following ACLT surgeries in mice using the von Frey filament assay (Fig. 1). Paw withdrawal threshold (PWT) was significantly reduced in the saline-treated ACLT mice when compared with the sham-operated controls (**p < 0.01), with a hind paw pressure of 2.3 g below baseline. At 4 weeks after treatment, both GE-P (300 mg/kg/day) and GE-L (300 mg/kg/day) decreased secondary allodynia (^##^*p* < 0.01 and ^##^*p* < 0.01, respectively) more than celecoxib (10 mg/kg/day) did (^#^*p* < 0.05) when compared with the saline group. The open-field test (OFT) revealed no significant differences in representative traveling patterns and total ambulatory distance (locomotion) between the low dose of GEG-treated, high dose GEG-treated, celecoxib-treated, and saline-treated mice (Fig. 2). The results also implied that ACLT-induced pain behavior in OA mice mainly resulted from tactile hypersensitivity of the paw, but motor dysfunction of the hidden legs was mostly unchanged between the sham-operated control and the experimental controls; the nerve pain could be relieved following treatment with the GEG extracts.

### GE extracts minimize osteolytic lesions in knee joints (radiographic evaluation)

**Fig. 3:**
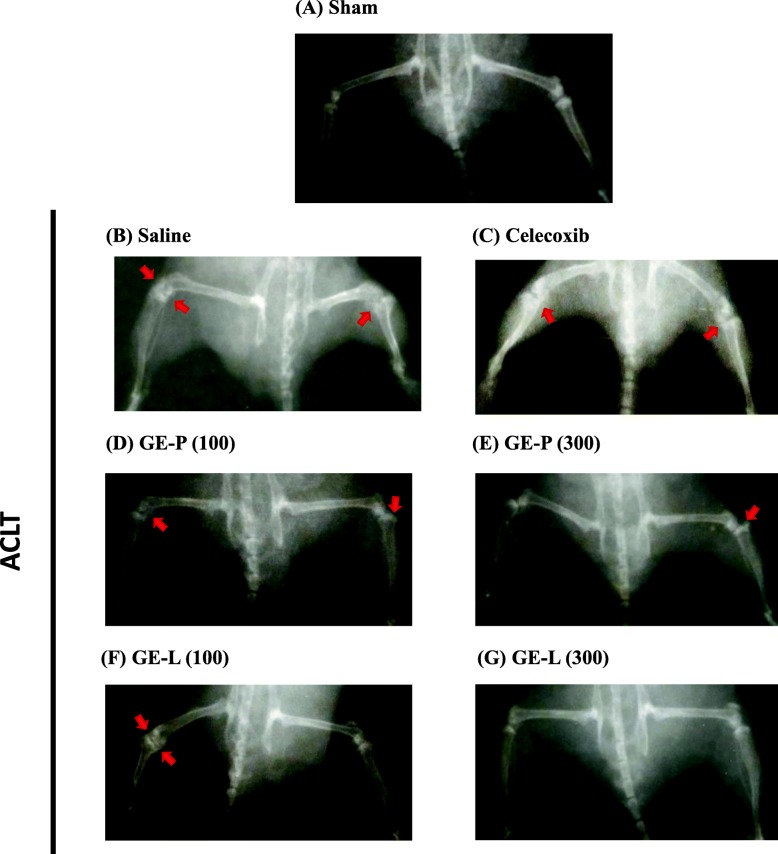
Determination of osteolytic lesions on knee joints in OA mice by radiography. On the 28th day before sacrifice, the mouse was placed in a lying position with the legs spread out in order to quantify subchondral bone thickening associated with post-traumatic OA by X-ray evaluation of the knee joints. The saline-treated group (ACLT, **b**) presented with osteolytic lesions and evidence of prominent bone formation extending into the soft tissue when compared with the vehicle control (sham, **a**). The architecture of both femur and tibia were relatively well preserved. Radiographs from the mice treated with GE-P (100 mg/kg/day, **d**), GE-P (300 mg/kg/day, **e**), GE-L (100 mg/kg/day, **f**), GE-L (300 mg/kg/day, **g**), and celecoxib (10 mg/kg/day, **c**) presented with areas of minimal subchondral thickness when compared with those in the saline group. The arrow denotes an area of increased subchondral bone thickness or prominent bone formation on the femur/tibia

Radiographic analysis was performed to observe the abnormal bone architecture and soft bone loss of between the femur and tibia subchondral bone. The plain radiographs in the sham-operated controls demonstrated relatively preserved architecture of the femur and tibia in mice 4 weeks after sham surgery (Fig. 3a), however. The saline-treated group can be detected radiographically, indicated by bony lesions and evident with prominent bone formation extending into the soft tissue (Fig. 3b). In ACLT-induced OA mice, these radiographic findings also confirmed that administration of GE-P (100 and 300 mg/kg/day) and GE-L (100 and 300 mg/kg/day) did significantly and dose-dependently (Fig. 3d–g) reduce the decrease in osteolytic lesions and bone spur formation compared to saline group (Fig. 3b). This radiographic appearance is quite similar to that of the sham-operated control, GE-L (300 mg/kg/day) had the better improving effect than celecoxib did (Fig. 3c) on the inhibition of the subchondral bone formation and the decrease of cartilage loss while improperly formed joints in ACLT-induced OA mice.

### GE extracts inhibit cytokines mRNA and protein expression of periarticular tissues

**Fig. 4:**
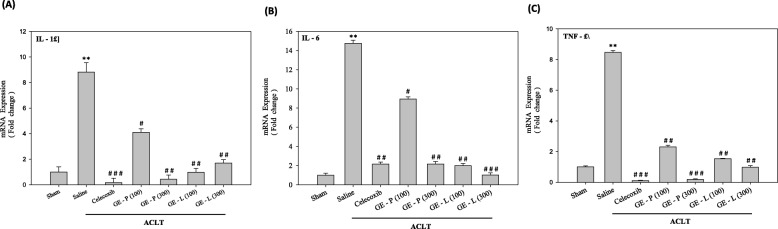
The mRNA expression of pro-inflammatory cytokines in the knees of the mice. Relative gene expression levels of inflammatory cytokines in the articular cartilages of the sham-operated or surgically destabilized mice that were treated with GE-P (100 and 300 mg/kg/day), GE-L (100 and 300 mg/kg/day), and celecoxib (10 mg/kg/day). The normalized gene expression levels are expressed as ratios of the copy number of mRNA to that of glyceraldehyde 3-phosphate dehydrogenase (GAPDH) cDNA. The mRNAs of the pro-inflammatory cytokines IL-1β (**a**), IL-6 (**b**), and TNF-α (**c**) were significantly elevated after saline-treatment (ACLT) when compared to the vehicle-treated mice, confirmed by quantitative real-time polymerase chain reaction (qRT-PCR). **p <0.01, significant difference when compared with the vehicle control (Sham). ^#^*p* < 0.05, ^##^*p* < 0.01, ^###^*p* < 0.001, significant differences when compared with the saline-treated (ACLT) group

**Fig. 5:**
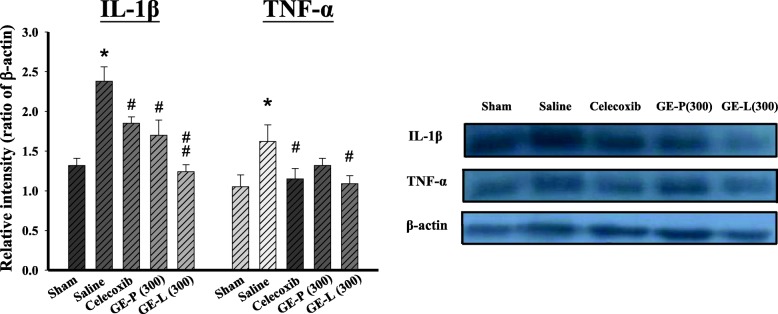
The proteins expression of pro-inflammatory cytokines in the knees of the mice. Relative protein expression levels of inflammatory cytokines in the articular cartilages of the sham-operated or surgically destabilized mice that were treated with GE-P (300 mg/kg/day), GE-L (300 mg/kg/day) and celecoxib (10 mg/kg/day). Expression levels of the IL-1β, TNF-α and β-actin proteins were determined by western blot and quantitated by microcomputer image device (MCID) image analysis. The β-actin levels were evaluated as a loading control, and the data are expressed as the IL-1β/β-actin and TNF-α/β-actin ratios, respectively. Each value represents the mean ± SE of three replicate experiments. *p < 0.01, significant difference when compared with the vehicle control (Sham). ^#^*p* < 0.05, significant differences when compared with the saline-treated (ACLT) group

In order to understand the mechanisms underlying the effects of GEG extracts on articular cartilage integrity, we examined the expression of genes encoding proteins with functions closely related to cartilage homeostasis. IL-1β, IL-6, and TNF-α are considered to be the main pro-inflammatory cytokines involved in the pathophysiology of OA. qPCR results showed that relative mRNA levels of IL-1β (Fig. 4a), IL-6 (Fig. 4b), and TNF-α (Fig. 4c) were significantly elevated in the saline-treated group (^**^p < 0.01) when compared with the sham-operated controls. GE-P (300 mg/kg/day) treatment significantly reduced the mRNA levels of IL-1β, IL-6, and TNF-α in the articular cartilage of the ACLT-induced OA mice when compared with the saline-treated mice (^##^*p* < 0.01, ^##^*p* < 0.01, and ^###^*p* < 0.001, respectively). Interestingly, GE-L (100 mg/kg/day) treatment also significantly decreased the mRNA levels of IL-1β, IL-6, and TNF-α when compared to celecoxib treatment in the ACLT-induced OA mice. Furthermore, IL-6 mRNA levels were significantly reduced in the GE-L (300 mg/kg/day) group (^###^*p* < 0.001 vs saline-treated) when compared with the celecoxib group (^##^*p* < 0.01 vs saline-treated) in the articular cartilages of the ACLT-induced OA mice. Consistently, a significant decrease in IL-1β and TNF-α protein expression was observed in the celecoxib treatment and GE-L (300 mg/kg/day) compared with the saline-treated mice (^#^*p* < 0.05, ^#^*p* < 0.05, and ^#^*p* < 0.05, ^##^*p* < 0.01, respectively). Interestingly, we also found that GE-L (300 mg/kg/day) treatment had dramatically decreased the levels of IL-1β and TNF-α expression than the GE-P (300 mg/kg/day) treatment did (^#^*p* < 0.05 and ^#^*p* > 0.05, respectively) (Fig. 5). These data suggested that the GE-L had an anti-inflammatory effect in vivo by decreasing the gene and protein expressions of proinflammatory cytokines.

### GE extracts improve cartilage degeneration in the knee joint (histopathologic evaluation)

**Fig. 6:**
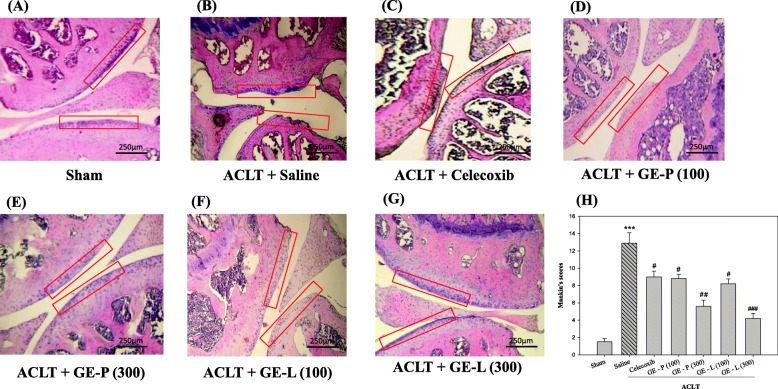
Morphological examination of the knee joints in OA mice (HE staining). All animals were sacrificed 28 days after the last treatment. A histopathological study of the cartilage and subchondral bone in ACLT-induced OA mice was performed after GE-P (100 mg/kg/day, **d**), GE-P (300 mg/kg/day, **e**), GE-L (100 mg/kg/day, **f**), GE-L (300 mg/kg/day, **g**), and celecoxib (10 mg/kg/day, **c**) treatment. In the normal group (sham, **a**), the articular cartilage tissue (red rectangle frame) remained relatively intact, with a well-preserved smooth cartilage surface and organized chondrocytes when compared with the saline group (ACLT, **b**), which presented with severe and extensive destruction of the cartilage in HE stained sections. Grading of the sections by a veterinary pathologist showed significant inflammation, synovial hyperplasia, and cartilage fissures along with disoriented and scattered dead chondrocytes in the saline group (sham). The Mankin score was evaluated for the grading of OA cartilage based on its structure and cell distribution in picture **h**. Damaged cartilage, disorganized chondrocyte clusters, and rough cartilage surfaces were seen in the ACLT-induced OA mice model. Original magnification, × 400. ***p < 0.001, significant difference when compared with the vehicle control (Sham). ^#^*p* < 0.05, ^##^*p* < 0.01, ^###^*p* < 0.001, significant differences when compared with the saline-treated (ACLT) mice

The beneficial effects of GEG extracts on articular cartilage and subchondral bone in OA mice were evaluated by staining the bone sections with HE and visualizing the cartilaginous tissue. HE staining (pale blue color) indicated the smooth surface, intact superficial layer, and normal chondrocyte population in the upper zone of the cartilage in the sham-operated control mice (Fig. 6a); conversely, erosion, clefting, chondrocyte degeneration, matrix changes, and typical chondrocyte clustering with apparent hypocellularity were noted in the saline group (Fig. 6b). For the histopathological classification of the severity of osteoarthritic lesions (denudation or deformation) in the cartilage, the Mankin score was used based on the OA grade levels of cartilage structure and cell distribution (Fig. 6h). Our results indicated that similarly to that of the celecoxib group (Fig. 6c, h, ^#^*p* < 0.05), the administration of 100 and 300 mg/kg/day of GE-P and GE-L attenuated cartilage destruction, with uneven surfaces and chondrocyte degeneration in the superficial layer in a dose-dependent manner (Fig. 6d–g). The Mankin scores were significantly reduced (^#^*p* < 0.05, ^##^*p* < 0.01 and ^#^*p* < 0.05, ^###^*p* < 0.001) when compared with the saline-treated group (Fig. 6b, h, ^***^p < 0.001 vs sham-operated control).

**Fig. 7:**
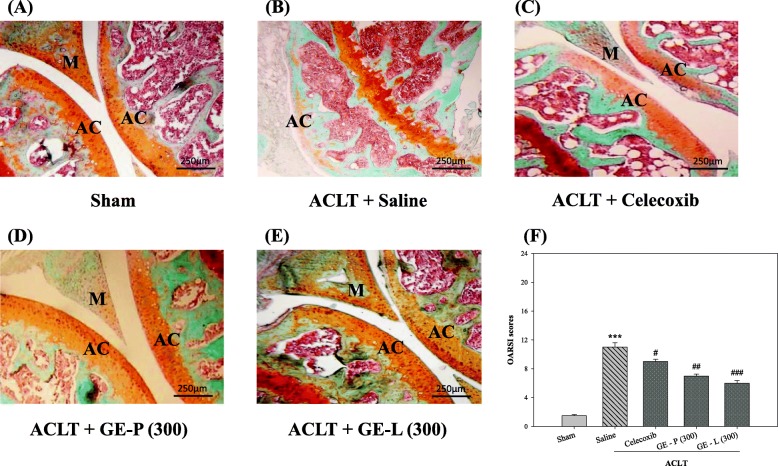
Histological changes in the knee joints (Safranin-O/fast green stain). As described previously, the histopathological appearances of the articular cartilage in ACLT-induced OA mice were examined after GE-P (300 mg/kg/day, **d**), GE-L (300 mg/kg/day, **e**), and celecoxib (10 mg/kg/day, **c**) treatment. Safranin-O/fast green staining (orange-red color indicates proteoglycan levels) in sagittal sections showing the main architecture (meniscus and cartilage) of the knee joint. Histological analysis using Safranin-O/fast green stain showing osteoarthritic changes in the articular cartilage of saline-treated mice (ACLT, **b**), whereas the knee joints of the vehicle controls (sham, **a**) showed no degenerative changes in the cartilage (**a**). Original magnification, × 400. Osteoarthritis Research Society International (OARSI) scores revealed less cartilage degeneration in the ACLT groups when compared with the other groups, indicating that GE-P and GE-L treatment reduced the extent of cartilage damage similar to celecoxib (**c**) treatment. Original magnification, × 400. All OARSI score statistics are shown in picture **f**. ***p < 0.001, significant difference when compared with the vehicle control (sham). ^#^*p* < 0.05, ^##^*p* < 0.01, ^###^*p* < 0.001, significant differences when compared with saline-treated (ACLT) mice. M, meniscus; AC, articular cartilage

We next determined the efficacy of Guilu Erxian Glue (GEG) extracts on ACLT-induced OA progression, the structural integrity of the articular cartilage was examined by microscopy after Safranin-O staining and OARSI evaluation. Safranin-O staining (a sensitive indicator of proteoglycan content) revealed serious OA lesions in the cartilage and meniscus in the saline-treated group 28 days after ACLT in mice; the mice exhibited moderate pathological osteoarthritic changes characterized by degeneration of articular cartilage, including proteoglycan loss, cartilage fibrillation, cartilage erosion, and an average OARSI of 10.7 ± 0.9 score (Fig. 7b, f, ^***^p < 0.001), compared to sham-operated control (Fig. 7a, f). In contrast, the cartilage of knee joints in GE-P (300 mg/kg/day) and GE-L (300 mg/kg/day)-treated mice exhibited less Safranin-O loss, no cartilage erosion, and significant cartilage degradation with a significantly lower OARSI score (6.7 ± 0.5 and 5.8 ± 0.4, respectively; ^##^*p* < 0.01, ^###^*p* < 0.001) compared to that in saline-treated group (Fig. 7d–f). Moreover, Safranin-O staining and OARSI evaluation demonstrated sufficient articular chondrocytes, retention of proteoglycan, and decreased thickness of calcified cartilage zone in GE-P (300 mg/kg/day) and GE-L (300 mg/kg/day)-treated mice relative to celecoxib group (Fig. 7c, f). Collectively, these results indicate that GE-G extracts would attenuate cartilage degeneration in OA development.

## Discussion

OA is a widespread chronic joint disease characterized by articular cartilage destruction accompanied with pain and disability [32, 33]. In the present study, we used a post-traumatic OA mouse model to test whether GE-G could slow the progression of OA and relieve both pain as well as OA-associated inflammation. The present study compared the anti-nociceptive, anti-inflammatory, and anti-arthritic activities of GE-P and GE-L prepared from GE-G extracts in ACLT-induced OA mice. The major findings of this study included inhibition of paw/joint pain, prevention of cytokine-induced inflammatory response, and suppression of certain osteoarthritis parameters (such as narrowing of joint space and osteophyte formation) by both GEG extracts. Moreover, the results demonstrate that both GE-P and GE-L exert similar effects on articular cartilage degeneration and subchondral bone deterioration when compared with celecoxib, and present with substantially lower Mankin and OARSI scores in the ACLT-induced OA mice.

Pain is the chief complaint of OA patients; however, due to the nature of the clinical studies and the limitation of the animal studies conducted so far, only a few have linked functional impairment and behavioral changes to cartilage loss and histopathology in OA animal models [34–36]. The findings of the present study demonstrate that GE-P and GE-L exhibited anti-nociceptive properties against mechanical stimuli in the paw, consistent with a state of chronic pain without causing motor impairment. Thus, we believe that the GEG extracts may be used as a clinically relevant aid in the treatment of chronic pain, possibly by acting as a good replacement for celecoxib during OA development. Recent reports have suggested that several TCMs are currently available for the treatment of chronic pain, especially neuropathic pain, which may have clinical relevance and open new possibilities for the development of new anti-hyperalgesic and anti-arthritic agents [37, 38].

OA is the most prevalent disease of the articular joints and is characterized by joint pain, narrowing of the joint space on the X-ray, and loss of joint function through progressive cartilage degradation and intermittent synovial inflammation [39, 40]. Compelling studies report the presence of empty lacunae and hypocellularity in the cartilage with OA progression, suggesting that chondrocyte cell death occurs and participates in OA development [41, 42]. In the current study, radiographic analyses of the knee joints confirmed that administration of GE-P (100 and 300 mg/kg/day) and GE-L (100 and 300 mg/kg/day) significantly and dose-dependently attenuated the narrowing of the joint space, osteophyte formation, and other features of osteoarthritis in the ACLT-induced OA mice. Meanwhile, nociception in the knee is complex, and the nociceptive stimuli are related to but fundamentally different from those producing cartilage loss [43]. As indicated in a previous study, daily treatment with celecoxib does not prevent cartilage degradation or osteophyte formation during OA development in the mouse model [44]. Consistently, significant inhibition of joint space narrowing and osteophyte formation was achieved with GE-L (300 mg/kg/day) treatment. These favorable effects were confirmed histologically in the same groups of animals, thus revealing the protective action of GE-P (300 mg/kg/day) and GE-L (300 mg/kg/day) against destruction and degeneration of cartilage in the mouse OA model.

Synovial inflammation is a frequently observed phenomenon in osteoarthritic joints and contributes to the pathogenesis of OA by the formation of various catabolic and pro-inflammatory mediators, which alter the balance between cartilage matrix degradation and repair [45]. Secreted inflammatory molecules such as pro-inflammatory cytokines are among the critical mediators of the disturbed processes implicated in OA pathophysiology [46]. Recent evidence has implicated cytokines including IL-1, IL-6, and TNF-α in the promotion of articular cartilage extracellular matrix protein degradation; they are also known to synergize with other cytokines to amplify and accelerate cartilage destruction [47, 48]. Most importantly, many of these cytokines have been implicated in causing synovial tissue activation, damage to subchondral bone and alterations in cartilage homeostasis in spontaneously occurring or surgically induced animal models of OA [49, 50]. The primary role of these cytokines is to modulate the expression of matrix metalloproteinases and cartilage extracellular matrix (ECM) proteins; in addition, they have been implicated in the development OA and could be therapeutically targeted in the future [51]. Interleukin-1 beta (IL-1β) is a potent pro-inflammatory cytokine that is capable of inducing chondrocytes and synovial cells to synthesize matrix metalloproteinases (MMPs), and acts as a key mediator of the degeneration of articular cartilage in OA [52]. IL-1β could also play a role in the early progression or initiation of OA as evidenced in many in vitro studies [53]. Induction of IL-1β expression in the TMJs of adult mice led to pathologic development, dysfunction, and related pain in the joints [54, 55].

Tumor necrosis factor alpha (TNF-α) is generally considered to be involved in the dysregulation of bone and cartilage remodeling in chondrodestructive diseases, especially osteoarthritis, in several in vitro and in vivo models [56, 57]. Recently, oral consumption of a hydrolyzed type 1 collagen preparation has been reported to reduce pain in human OA, and the supplemented mice also displayed reduced synovial hyperplasia that paralleled a reduction in TNF-α mRNA suggesting an anti-inflammatory effect [58]. Furthermore, the use of IL-1 and/or TNF-α inhibitors in experimental models of inflammatory arthritis and OA has provided strong support for the role of IL-1 in the regulation of catabolic events and inflammatory processes in degenerative joint diseases [59]. It is generally accepted that IL-1 is the pivotal cytokine during the early and late stages, while TNF-α is primarily involved during the onset of arthritis, and IL-6 is released during the inflammatory process in an OA joint [60]. In chondrocytes and cartilage explants, IL-6 treatment can stimulate chondrocyte calcification and reduce proteoglycan content with increased production of MMP-3 and MMP-13 [61]. IL-6 gene knock out in male mice resulted in the development of advanced OA suggesting its role as a crucial mediator in the biomechanical control of cartilage destruction and bone remodeling in OA [62, 63]. Our qPCR results showed that the mRNA levels of IL-1β, IL-6, and TNF-α in the articular cartilage were partially reversed by GE-L (100 mg/kg/day) treatment similar to the effects of celecoxib (10 mg/kg/day), whereas GE-L (300 mg/kg/day) treatment exerted maximum inhibition on the IL-6 mRNA levels resulting in amelioration of OA progression in the mice. In this study, a significant decrease in IL-1β and TNF-α protein expression was observed in the celecoxib treatment and GE-L (300 mg/kg/day) more than the GE-P (300 mg/kg/day) treatment did as compared to the saline-treated mice. These data suggested that the GE-L had an anti-inflammatory effect in vivo is consistent with those from previous studies where treatment with celecoxib (5 mg/kg) was effective in decreasing the elevated levels of IL-1β and TNF-α, inhibiting synovial thickening and balancing MMPs levels, thus resulting in the preservation of ECM in a posttraumatic OA mouse model [64, 65].

Several recent experimental studies performed in animal models of OA sustained the previously held view that the magnitude of behavioral changes was directly correlated with higher OARSI histological scores of OA, synovitis in the knee joints, cartilage volume loss, and osteophyte formation [66, 67]. Loss of articular cartilage is a crucial event in OA and is characterized by a disturbance in the regulation of synthetic (anabolic) and resorptive (catabolic) activities of the resident chondrocytes, which results in net loss of the cartilage matrix components and deterioration in the structural and functional properties of the cartilage [68, 69]. In the present study, histological analysis demonstrated progressive joint degeneration, as measured by a modified Mankin scale and an OARSI score, with significantly less cartilage destruction and proteoglycan loss in the articular cartilage after GE-L (300 mg/kg/day) administration compared to that of GE-P (300 mg/kg/day) while GE-L (100 mg/kg/day) and GE-P (100 mg/kg/day) had the same treatment effectiveness. Observations from HE and Safranin-O staining suggest that GE-P (300 mg/kg/day) rather than GE-L (300 mg/kg/day) can protect the mice chondrocytes from degeneration and normalize the altered cartilage matrix remodeling/degradation via catabolic reactions caused by the cytokines (IL-1β and TNF-α). These morphological data also suggest that the potency of GE-L is remarkably similar to GE-P, even the high dose (300 mg/kg/day) of GE-L can exert greater chondroprotective effect in an attempt to reduce inflammation in vivo, alleviates synovitis, retards the senescence of chondrocytes and suppresses structural changes while reducing the number of catabolic enzymes in the knee cartilage in OA.

## Conclusions

This study demonstrates that the anti-arthritic effects of GE-L (a novel substitute for GE-P, which is not convenient for oral administration) were better than those of celecoxib. Dose-dependent increases in the narrowing of joint space, and in cartilage area, as well as the proteoglycan matrix, are blocked early during the osteoarthritic process and sustained through the course of the disease, partially through nociceptive and inflammatory responses. These results may highlight the need to develop new therapeutic TCM in order to improve the management of patients with OA, which currently lack general therapeutic principles.

## Abbreviations

ACLT: Anterior cruciate ligament
COX-2: Cyclooxygenase-2
ECM: Extracellular matrix
GAPDH: Glyceraldehyde 3-phosphate dehydrogenase
GEG: Guilu Erxian Glue
GE-L: Guilu Erxian Liquid
GE-P: Guilu Erxian Paste
HE: Hematoxylin-eosin
IL-1β: Interleukin-1β
MMPs: Matrix metalloproteinases
MV: Mean velocity
NSAIDs: Non-steroidal anti-inflammatory drugs
OA: Osteoarthritis
OARSI: Osteoarthritis Research Society International
OFT: Open-field test
PWT: Paw withdrawal threshold
qPCR: Quantitative real-time PCR
SD: Standard deviation
TCM: Traditional Chinese medicine
TNF-α: Tumor necrosis factor-alpha
